# The associations between organophosphate esters and urinary incontinence in the general US population

**DOI:** 10.1007/s11356-021-14153-5

**Published:** 2021-09-14

**Authors:** Mingjing He, Kun Jin, Shi Qiu, Xinyang Liao, Xiaonan Zheng, Zeyu Chen, Jianzhong Ai, Lu Yang, Zhongyuan Jiang, Dan Hu, Qiang Wei

**Affiliations:** 1 Department of Urology, Institute of Urology, West China Hospital, Sichuan University, Chengdu, Sichuan China; 2 Department of Clinical Research Management, West China Hospital, Sichuan University, Chengdu, Sichuan China

**Keywords:** Organophosphate esters (OPEs), Metabolites, Urinary incontinence, Women, NHANES

## Abstract

Organophosphate esters (OPEs) impact health in many ways. Since its relationship with urinary incontinence remains unknown, we aimed to explore their associations in the US general population. We combined the results of urine specimens test and self-reported urinary incontinence conditions from the National Health and Nutrition Examination Survey (NHANES) 2013–2014 among 2666 participants and then conducted linear regression and logistic regression to analyse associations between log2-transformed OPE concentrations and urinary incontinence. We found that 0.92% of men and 15.74% of women complained of mixed urinary incontinence (MUI). The concentrations of diphenyl phosphate (DPHP) were significantly correlated to MUI among women when treated as a continuous variable (adjusted odds ratio (OR) = 1.15; 95% confidence interval (CI), 1.01–1.31; *p* = 0.0369) and as a categorical variable (adjusted OR = 1.24; 95% CI, 1.03–1.49; *p* for trend = 0.0245), whereas no positive correlation was found in males. There were no significant associations between the other three OPEs: bis(2-chloroethyl) phosphate (BCEP), bis(1,3-dichloro-2-propyl) phosphate (BDCPP) and dibutyl phosphate (DBUP). The association of DPHP with an increased prevalence OR of MUI in women is a public health concern; future prospective studies are needed to explore its potential mechanism.

**Supplementary Information:**

The online version contains supplementary material available at 10.1007/s11356-021-14153-5.

## Introduction

Organophosphate esters (OPEs) are a group of synthetic chemicals that have been widely used as replacements for brominated flame retardants (Kemmlein et al. 2003) in construction materials, in furniture, and in the outer shells of electronics. They are also known as plasticisers when used in consumer products (e.g. food packages, beverage containers, children’s products and cosmetics) (Luo et al. 2020; Mendelsohn et al. 2016). According to national biomonitoring data, OPE metabolites are widespread in the USA, at least eight kinds were detected in most participants of a previous study (Jayatilaka et al. 2019), as well as in common dining table food (e.g. meat, vegetables, eggs, dairies and cereals) (Ding et al. 2018). Since OPEs are metabolism-disrupting compounds, exposure to these may affect the thyroid hormone pathway, which is critical for the growth and development of birds and humans. The affected metabolites can alter sex hormone balance and decrease semen quality in men (Farhat et al. 2013; Meeker and Stapleton 2010; Zhang et al. 2016).

Urinary incontinence, one of the most prevalent health concerns confused in adults, contributes to decreased quality of life, especially in older women (Vaughan and Markland 2020). A representative study done in the USA (Dooley et al. 2008) reported that 2,098 (49.6%) of the 4229 women suffer from urinary incontinence symptoms. Among them, 50% reported stress urinary incontinence (SUI), 34.3% reported mixed urinary incontinence (MUI) and 15.9% reported urge urinary incontinence (UUI). The symptoms or signs of MUI include involuntary loss of urine associated with SUI and UUI. These are both affected by lifestyle and urinary habits (Haylen et al. 2010); thus, the mechanism of MUI is complicated. OPEs are metabolism-disrupting compounds which affect the biological activity; we hypothesize that environmental hazardous pollutants, such as OPE metabolites, could increase the risk for MUI. However, no study has provided insight into the connection between the two.

In the present study, we detected several common OPEs in 1525 participants from the National Health and Nutrition Examination Survey (NHANES) 2013–2014. We aimed to determine the correlation between OPE exposure and MUI in the US general population.

## Materials and methods

### Study population

The NHANES has been conducted continuously since 1999 by the National Center for Health Statistics (NCHS) at the Centers for Disease Control and Prevention (CDC). This survey is rigorously and comprehensively conducted to determine the current health and nutritional status of the US general population through interviews and physical examinations. To find the possible link between OPE metabolite exposure and MUI, we analysed data from the 2013–2014 NHANES. Although incontinence data from NHANES 1999–2018 are available, flame retardant metabolite data were only available for random one-third subset of NHANES participants 6 years old and older during 2013–2014. The analytic cohort was restricted to participants with collected urine specimens (*n* = 2666) who were randomly chosen by the NCHS for OPE analysis, and then participants with complete data on the following four widely distributed OPEs were included: diphenyl phosphate (DPHP), bis(2-chloroethyl) phosphate (BCEP), bis(1,3-dichloro-2-propyl) phosphate (BDCPP) and dibutyl phosphate (DBUP). Pregnant women (*n* = 12) were excluded from the analysis since pregnancy may affect urinary function. We also excluded participants with incomplete urinary incontinence data (*n* = 507) or covariate information (*n* = 87). After these exclusions, there remained a cohort of 1,525 as analytic samples.

### Outcomes and measurement of predictors

The primary outcome, MUI, was determined by participants’ response to these questions: (1) ‘During the past 12 months, have you leaked or lost control of even a small amount of urine with an activity like coughing, lifting or exercise?’; (2) ‘During the past 12 months, have you leaked or lost control of even a small amount of urine with an urge or pressure to urinate and you couldn’t get to the toilet fast enough?’ and (3) ‘How frequently does this occur?’ The following questions were ulteriorly used to assess the presence of incontinence: (1) ‘Have you leaked or lost control of even a small amount of urine with an activity like coughing, lifting or exercise?’ and (2) ‘Have you leaked or lost control of even a small amount of urine with an urge or pressure to urinate and you could not get to the toilet fast enough?’ Mixed incontinence was defined as answering yes to both questions. The urine specimens were collected and tested by staff members of NHANEs, we selected four urinary biomarkers: DPHP, BCEP, BDCPP and DBUP from them for analysis.

We performed the analysis using Empower Stats, version 2.0. Statistical analysis was conducted following the NHANES analytic guidelines (‘National Health and Nutrition Examination Survey. Analytic guidelines, 1999–2010’, 2013). We defined five race/ethnicity groups: (1) Mexican American, (2) non-Hispanic Black, (3) non-Hispanic White, (4) other Hispanic and (5) other races based on self-reported data. We calculated the geometric means (GMs) and distribution percentiles for the concentrations of OPEs (in μg/L), which were log2-transformed because they were right-skewed distributed.

### Statistical analysis

We performed the analysis using Empower Stats, version 2.0. Statistical analysis was conducted following the NHANES analytic guidelines (‘National Health and Nutrition Examination Survey. Analytic guidelines, 1999–2010’, 2013). We defined five race/ethnicity groups: (1) Mexican American, (2) non-Hispanic Black, (3) non-Hispanic White, (4) other Hispanic and (5) other races based on self-reported data. We calculated the geometric means (GMs) and distribution percentiles for the concentrations of OPEs (in μg/L), which were log2-transformed because they were right-skewed distributed.

We calculated the descriptive statistics for the concentrations of OPE metabolites detected among children and adults, including weighted GMs, percentiles and ranges. OPE metabolites with a detection frequency (DF) ≥ 80% (i.e. DPHP, BDCPP, BCEP and DBUP) were modelled as continuous log2-transformed-independent variables in separate models as previously specified. To increase statistical power and precision of effect estimates, these concentrations were replaced below the LOD with LOD/√2 for OPE metabolites detected in ≥ 80% of the study population (Hornung and Reed 1990; Cole et al. 2009). We used sampling weights as well as strata and primary sampling units to account for the selective bias, nonresponse adjustment and oversampling of certain subpopulations. To adjust the urine dilution, we used a traditional creatinine adjustment, wherein the urinary chemical concentration was divided by the urinary creatinine concentration. Furthermore, to control potential confounding caused by compromised kidney function on creatinine excretion, we employed a ‘novel creatinine adjustment’ of covariate-adjusted standardization (Bulka et al. 2017; O’Brien et al. 2016).

*T*-test and chi-square test were used to compare the means and proportions of baseline characteristics. Multivariable logistic regression analysis was used to estimate the impact of OPE metabolites (continuous variables) on MUI. Multivariate models included the crude model, minimally adjusted model (age, race, physical activity, energy intake, education level, marital status, alcohol intake, body mass index (BMI), cardiovascular disease (CAD) score and poverty–income ratio (PIR) were adjusted) and fully adjusted model II (log10-transformed urinary creatinine concentrations was further adjusted). Estimates from these linear and logistic regression analyses were considered as the main results. A few additional analyses were also conducted. First, we used tertiles of OPE metabolites as ordinal categorical variables (first to third, setting the first tertile as reference) to examine potential trends in the association. Second, to account for the non-linearity of the MUI, we conducted smooth curve fitting (penalised spline method) and used a weighted generalised additive model. All statistical analyses were conducted using Empower Stats, version 2.0. A two-sided *p* value of < 0.05 was considered statistically significant.

## Results

### Participant characteristics

The current study enrolled a total of 1,525 participants: 749 men (49.04%) and 776 women (50.96%) with weighted mean ages of 46.19 ± 16.88 and 45.72 ± 15.59 years old, respectively. Notably, 66.41% of the participants were non-Hispanic Whites, whereas 63.05% of them reported to be married or living with a partner. MUI complaints were found in 0.92% of men and 15.74% of women. The participants’ physical activity, education level and other baseline characteristics are presented in Table 1.

**Table 1:** Baseline characteristics of the study population from the 2013–2014 NHANES cohort

	Total	Men	Women	*p* value
	(*n* = 1525)	(*n* = 749)	(*n* = 776)	
Age	45.72 ± 15.58	46.19 ± 16.88	45.72 ± 15.59	0.0041
Energy intake (kcal)	2188.71 ± 1007.00	2518.79 ± 1108.90	2188.71 ± 1007.01	<0.0001
Creatinine of urine (mg/dL)	419.30 ± 176.27	135.53 ± 80.28	419.30 ± 176.28	<0.0001
Race (%)				0.4755
Mexican American	9.2	10.37	11.54	
Other Hispanic	5.96	5.91	5.86	
Non-Hispanic White	66.41	65.37	64.33	
Non-Hispanic Black	10.52	9.9	9.28	
Other races	7.91	8.45	8.99	
Physical activity (%)				0.0046
Less than moderate	44.72	41.28	37.84	
Moderate	10.66	9.84	9.02	
Vigorous	44.62	48.87	53.12	
Education level (%)				0.1858
Less than high school	13.26	14.22	15.18	
High school or GED	20.98	22.46	23.94	
Above high school	65.76	63.32	60.88	
Marital status (%)				<0.0001
Married or living with partner	63.05	64.72	66.39	
Living alone	36.95	35.28	33.61	
Alcohol intake (%)				<0.0001
None	71.56	67.2	62.84	
Moderate	9.22	14.02	18.82	
Heavy	19.22	18.78	18.34	
BMI (%)				<0.0001
< 25	30.66	27.24	23.82	
≤ 25 and< 30	31	36.91	42.82	
≥ 30	38.34	35.85	33.36	
CAD score (%)				0.0363
0	63.4	64.62	62.22	
1	23.34	20.75	25.84	
2	13.26	14.63	11.94	
PIR (%)				0.7985
< 1.3	21.91	21.39	20.87	
≤1.3 and < 3.5	38.16	37.88	37.6	
≥ 3.5	39.93	40.73	41.53	
MUI (%)				<0.0001
No	91.53	99.08	84.26	
Yes	8.47	0.92	15.74	

Mean ± SD for: age, energy intake, creatinine of urine (mg/dL)

### OPE metabolite concentrations

Based on the original results of the NHANES 2013–2014 investigation, only four chemicals (DPHP, BDCPP, BCEP and DBUP) were detected at levels over 75% among all the included monitoring OPE metabolites. Table 2 illustrates their GMs, distribution percentiles, LODs and maximum values of concentrations. After adjusting for urine dilution using the log10-transformed urinary creatinine concentrations adjustment, BDCPP had the highest GM, followed by BCEP, DPHP and then DBUP (0.81, 0.80, 0.66 and 0.42 ng/mL, respectively; see Table 2).

**Table 2:** Detection and distribution of OPE metabolites in urine of the general US population (*n* = 1525)

		GM (95% CI)	LOD (μg/L)	p25	p50	p75	Max
DPHP	Unadjusted	0.71 (0.64 0.78)	− 3.1844	− 1.6439	− 0.4941	0.5160	6.9773
	Adjusted I	0.69 (0.62 0.77)	− 4.6543	− 1.3001	− 0.6004	0.3446	6.8138
	Adjusted II	0.66 (0.60 0.73)	− 3.4087	− 1.5237	− 0.5347	0.4525	6.8594
BDCPP	Unadjusted	0.88 (0.81 0.96)	− 3.6439	− 1.8365	− 0.5146	0.8074	6.4741
	Adjusted I	0.65 (0.59 0.71)	− 4.3219	− 1.4792	− 0.5236	0.5146	6.0845
	Adjusted II	0.81 (0.74 0.89)	− 3.8799	− 1.7149	− 0.5191	0.6878	6.2913
BCEP	Unadjusted	0.81 (0.72 0.92)	− 4.0589	− 2.8365	− 1.4739	− 0.2345	6.7814
	Adjusted I	0.74 (0.64 0.85)	− 5.3923	− 2.4150	− 1.5146	− 0.4361	5.9153
	Adjusted II	0.80 (0.70 0.91)	− 4.2831	− 2.6989	− 1.4948	− 0.3159	6.4399
DBUP	Unadjusted	0.50 (0.32 0.76)	− 4.6439	− 3.8365	− 2.1844	− 1.5146	1.9672
	Adjusted I	0.49 (0.30 0.80)	− 6.5584	− 3.2345	− 2.3520	− 1.6323	2.9672
	Adjusted II	0.42 (0.27 0.67)	− 4.9645	− 3.7286	− 2.1687	− 1.5874	2.0770

Percentile knots were used for the log2-transformed OPE metabolite levels. The knots were located at the 25th, 50th and 75th percentiles; units are in μg/L. Adjusted model I for age, race, physical activity, energy intake, education level, marital status, alcohol intake, BMI, CAD score and PIR; adjusted model II for age, race, physical activity, energy intake, education level, marital status, alcohol intake, BMI, CAD score, PIR and log10-transformed urinary creatinine concentrations

### Association between OPE metabolite concentrations (continuous) and MUI

DPHP showed a significant association with MUI (adjusted model I for age, race, physical activity, energy intake, education level, marital status, alcohol intake, BMI, CAD score and PIR); a 1 ng/mL increase in DPHP was associated with 15% increased prevalence odds ratio (OR) for MUI (OR = 1.15; 95% confidence interval (CI), 1.02–1.31; *p* = 0.026). The results after adjustment for log10-transformed urinary creatinine concentrations (adjusted model II) were consistent with model I (OR = 1.15; 95% CI, 1.01–1.31; *p* = 0.0369). However, after dividing the participants by gender, only the women group retained the consistent results (OR = 1.15; 95% CI, 1.01–1.31; *p* = 0.0322 in adjusted model I and OR = 1.16; 95% CI, 1.01–1.33; *p* = 0.0376 in adjusted model II). In contrast, no statistically significant association was observed between DPHP and MUI in the individual men group. No positive association was observed between BDCPP, BCEP, DBUP and MUI among all participants (Table 3). In the sensitivity analyses, the main results did not change either when we included delivery conditions in the confounding factors (*p* < 0.05 for DPHP and *p* > 0.05 for BDCPP, BCEP and DBUP; see Supplementary Table 1).

**Table 3:** Multivariate logistic regression of MUI with and without adjustment

OPE		Total		Men		Women	
		OR (95% CI)	*p* value	OR (95% CI)	*p* value	OR (95% CI)	*p* value
DPHP	Adjusted I	1.15 (1.02, 1.31)	0.0260	1.05 (0.66, 1.67)	0.8224	1.15 (1.01, 1.31)	0.0322
	Adjusted II	1.15 (1.01, 1.31)	0.0369	0.95 (0.53, 1.68)	0.8505	1.16 (1.01, 1.33)	0.0376
BDCPP	Adjusted I	0.97 (0.86, 1.11)	0.6956	0.88 (0.60, 1.30)	0.5310	0.99 (0.86, 1.13)	0.8355
	Adjusted II	0.94 (0.82, 1.07)	0.3632	0.79 (0.48, 1.27)	0.3267	0.95 (0.83, 1.09)	0.4772
BCEP	Adjusted I	1.05 (0.93, 1.17)	0.4526	0.76 (0.51, 1.14)	0.1822	1.07 (0.95, 1.21)	0.2528
	Adjusted II	1.03 (0.91, 1.16)	0.6290	0.69 (0.42, 1.15)	0.1553	1.06 (0.94, 1.20)	0.3617
DBUP	Adjusted I	0.95 (0.81, 1.11)	0.4982	1.24 (0.72, 2.15)	0.4345	0.92 (0.78, 1.09)	0.3187
	Adjusted II	0.97 (0.83, 1.15)	0.7556	1.01 (0.54, 1.87)	0.9798	0.96 (0.81, 1.14)	0.6345

Adjusted model I for age, race, physical activity, energy intake, education level, marital status, alcohol intake, BMI, CAD score and PIR; adjusted model II for age, race, physical activity, energy intake, education level, marital status, alcohol intake, BMI, CAD score, PIR and log10-transformed urinary creatinine concentrations

### Association between OPE metabolite concentrations (categorical) and MUI

To further assess the relationship between OPE metabolite concentration and MUI, we converted DPHP concentrations from continuous variables to categorical variables (in tertiles). In adjusted model I, the ORs in the higher tertiles (T2, T3) when compared to the lowest tertile (T1) tended to be higher among all participants (OR = 1.24; 95% CI, 1.03–1.48; *p* for trend = 0.0206). After adjustment for log10-transformed urinary creatinine concentrations (adjusted model II), the OR remained significant (OR = 1.24; 95% CI, 1.03–1.49; *p* for trend = 0.0245). Similarly, consistent results were found in the women group (OR = 1.23; 95% CI, 1.02–1.49; *p* for trend = 0.0283 in adjusted model I and OR = 1.25; 95% CI, 1.03–1.52; *p* for trend = 0.0262 in adjusted model II). However, no significant OR was found in the men group (see Supplementary Table 2). We did not observe significant outcomes in the other three OPEs (BDCPP, BCEP and DNBP; see Supplementary Tables 3, 4 and 5).

## Discussion

In the present study, we used the 2013–2014 NHANES database, which is considered to be representative of the population of the USA, to explore the correlations between MUI and four OPEs. More women were found to complain of MUI compared to men. The GM concentrations of DPHP were relative to MUI among women when treated both as a continuous or categorical variable. This association remained significant after adjustment for potential confounding factors. However, there were no significant associations between the other three OPEs (BDCPP, BCEP and DBUP).

Other health problems linked to OPEs have been reported in many previous studies. However, to the best of our knowledge, this is the first study that explored the associations between OPE metabolites and urinary incontinence. For example, an in vitro investigation showed that OPE toxicity led to higher incidence of mortality and malformation in zebrafish embryos (Tran et al. 2020; Liu et al. 2012). Xu et al. reported that tri-ortho-cresyl phosphate (TOCP), a kind of OPE, leads to hepatocellular injury with elevation of serum aminotransferase levels in mice (Xu et al. 2016). Similarly, Lee et al. also demonstrated that DPHP is significantly associated with uterine fibroids (Lee et al. 2020). Moreover, Meeker et al. found that triphenyl phosphate (TPP), another kind of OPE, may be associated with an increase in prolactin and decrease in semen quality in men (Meeker and Stapleton 2010). These previous studies, along with our additional evidence, suggest that OPE metabolites may pose a substantial public health hazard.

MUI can seriously affect one’s quality of life by taking the form of stress incontinence, urge incontinence or both (Irwin et al. 2010). SUI is the involuntary loss of urine with increased intra-abdominal pressure or physical exertion (e.g. coughing, jumping and physical exercise), whereas UUI is caused by irritation or loss of neurologic control over bladder contractions. The existing literature identifies much more complex causes of MUI, because it involves striated muscle atrophy, oestrogen status and inappropriate sensory and motor innervation of the bladder. Some ongoing studies are even assessing the role of plasma glucose in MUI. An investigation regarding diabetes and urinary incontinence in 1461 adult women suggested that women with diabetes or impaired fasting glucose had a higher prevalence of MUI than women with normal fasting glucose (Brown et al. 2006).

Fang et al. reported that some OPE metabolites can activate the peroxisome proliferator-activated receptor (PPAR), a master nuclear receptor that regulates lipid metabolism and adipocyte gene expression and may be a key factor for obesity (Fang et al. 2015). Wang et al. found that TPP exposure causes an increase in BMI, liver weight and hepatic steatosis by altering the expression of genes related to lipid metabolism (Wang et al. 2019). Furthermore, a study of 769 women was able to identify obesity as an important risk factor for urinary incontinence in Korean women; obesity can impact the vascular system of the pelvic floor, thus affecting detrusor and sphincter muscle functions. In addition, segmental adiposity can increase abdominal diameters as well as intra-abdominal and bladder pressure, resulting in fatigue, weakness of the pelvic floor muscles and impaired bladder control (Han et al. 2006). These connections pose an important question: is it possible that OPE metabolites increase the risk of MUI by regulating the expression of genes related to obesity?

OPE metabolites are regarded as endocrine-disrupting compounds (EDCs), which mimic hormones in the endocrine system and disrupt the physiologic function of endogenous hormones, as well as interfere with energy homeostasis, lipid metabolism and insulin sensitivity. Many EDCs exhibit oestrogenic or anti-androgenic activity; they can either modify the synthesis or actions of oestradiol and testosterone or interfere with their respective hormone receptors (Giwercman and Giwercman 2011). After a review of the literature, we suspect that OPE metabolites increase the risk of MUI by decreasing testosterone levels. In a rat study, pelvic floor muscle atrophy improved after administration of testosterone in an experimentally induced SUI model; this was suspected to be due to the anabolic effect of testosterone on the pelvic floor muscles (Cody et al. 2012) (Mammadov et al. 2011). Moreover, Michelle et al. explored the relationship of serum testosterone levels and urinary incontinence in women and found a positive correlation between low serum testosterone and SUI and MUI risks. In another study, testosterone has been shown to bind androgen receptors located in the pelvic floor muscle and fascia, thus further changing the function of the levator ani (Kim and Kreydin 2018). On average, testosterone levels in adult men are approximately 7–8 times as great as in adult females, because their more pronounced metabolism causes the daily production to be approximately 20 times greater in males. This is programmed to diminish with age, particularly in postmenopausal women, who are also more sensitive to testosterone (Southren et al. 1965; Torjesen and Sandnes 2004). Perhaps this may be a potential cause for the higher prevalence of urinary incontinence in women.

Our study has the following limitations. First, the laboratory studies of OPE metabolites and urinary incontinence were limited. Because we used a cross-sectional design, even if it was adequately representative of the US population, we cannot conclude a causal relationship between MUI and OPEs without more experimental results or direct evidence. Second, urine was only collected once, and thus the 24-h changes in the components of urine change may have been missed. In addition, a single measurement of OPE could not represent long-term exposure because of the short biological half-lives of OPEs. Finally, urinary incontinence was measured by answers to questions, the results of which could be influenced by subjective factors, unless we use the urodynamic test to confirm the diagnosis of urinary incontinence.

## Conclusion

To our knowledge, this is the first study focusing on the association between urinary incontinence and OPEs among the general US adult population. DPHP was found to be associated with an increased OR for MUI in women, and some directions for further study were included. Due to the public health concern of OPE metabolite exposure, future prospective research is warranted to explore its potential mechanisms.

## Supplementary Information

ESM 1 (DOCX 2450 kb)

## Data Availability

All data of the current study are in public and available free of charge in the NHANES repository (https://wwwn.cdc.gov/nchs/nhanes/Default.aspx).

## Abbreviations

MUI: Mixed urinary incontinence
UUI: Urge urinary incontinence
SUI: Stress urinary incontinence
NCHS: National Centre for Health Statistics
NHANES: National Health and Nutrition Examination Survey
DPHP: Diphenyl phosphate; BCEP, bis(2-chloroethyl) phosphate
BDCPP: Bis(1,3-dichloro-2-propyl) phosphate
DBUP: Dibutyl phosphate
TOCP: Tri-ortho-cresyl phosphate
PPAR: Peroxisome proliferators-activated receptor
EDCs: Endocrine disrupting compounds
PIR: Poverty–income ratio
BMI: Body mass index
CAD score: Cardiovascular disease score
GED: General educational development
DF: Detection frequency
GM: Geometric mean
LOD: Limit of detection
OR: Odds ratio
CI: Confidence interval
US: United States
