# Supplementary material for: The associations between organophosphate esters and urinary incontinence in the general US population
Source: Environ Sci Pollut Res Int. 2021 Sep 14;29(7):10400–7. doi: 10.1007/s11356-021-14153-5 (PMC8783883; doi:10.1007/s11356-021-14153-5)

**Supplementary table 1. Sensitivity analysis of odds of MUI after separately adjusting for delivery**

|  |  | OR (95%CI) | p value |
| --- | --- | --- | --- |
| DPHP | Adjust Id | 1.15 (1.01, 1.32) | 0.0321* |
|  | Adjust II | 1.16 (1.01, 1.33) | 0.0404* |
| BDCPP | Adjust Id | 0.99 (0.86, 1.13) | 0.8303 |
|  | Adjust II | 0.95 (0.83, 1.10) | 0.4861 |
| BCEP | Adjust Id | 1.07 (0.95, 1.21) | 0.2703 |
|  | Adjust II | 1.06 (0.93, 1.20) | 0.4023 |
| BDUP | Adjust Id | 0.92 (0.78, 1.09) | 0.3269 |
|  | Adjust II | 0.96 (0.81, 1.14) | 0.6311 |

Adjusted Id for age, race, physical activity, energy intake, education level, Marital status, alcohol intake, BMI, CAD score, PIR

PIR, log10-transformed urinary creatinine concentrations, delivery condition.

Adjusted II for age, race, physical activity, energy intake, education level, Marital status, alcohol intake, BMI, CAD score, PIR

PIR, log10-transformed urinary creatinine concentrations

**Supplementary table 2. Multivariate logistic regression of MUI by DPHP with and without adjustment**

|  |  | overall | |  | Men | |  | Women | |  |
| --- | --- | --- | --- | --- | --- | --- | --- | --- | --- | --- |
|  |  | OR (95%CI) | p value |  | OR (95%CI) | p value |  | OR (95%CI) | p value |  |
| Adjust I | T 1 | 1 |  |  | 1 |  |  | 1 |  |  |
|  | T 2 | 1.48 (0.90, 2.42) | 0.1218 |  | 1.22 (0.29, 5.25) | 0.7858 |  | 1.48 (0.88, 2.50) | 0.1390 |  |
|  | T 3 | 1.83 (1.10, 3.04) | 0.0197* |  | 1.39 (0.28, 7.00) | 0.6881 |  | 1.82 (1.07, 3.11) | 0.0270* |  |
|  | p for trend | 1.24 (1.03, 1.48) | 0.0206* |  | 1.12 (0.63, 1.99) | 0.6888 |  | 1.23 (1.02, 1.49) | 0.0283* |  |
| Adjust II | T 1 | 1 |  |  | 1 |  |  | 1 |  |  |
|  | T 2 | 1.53 (0.91, 2.55) | 0.1054 |  | 0.82 (0.16, 4.35) | 0.8189 |  | 1.53 (0.89, 2.63) | 0.1283 |  |
|  | T 3 | 1.85 (1.09, 3.13) | 0.0227* |  | 0.99 (0.15, 6.48) | 0.9927 |  | 1.89 (1.08, 3.30) | 0.0247* |  |
|  | p for trend | 1.24 (1.03, 1.49) | 0.0245* |  | 1.00 (0.51, 1.98) | 0.9956 |  | 1.25 (1.03, 1.52) | 0.0262* |  |

Adjusted I for age, race, physical activity, energy intake, education level, Marital status, alcohol intake, BMI, CAD score, PIR

Adjusted II for age, race, physical activity, energy intake, education level, Marital status, alcohol intake, BMI, CAD score, PIR, log10-transformed urinary creatinine concentrations

**Supplementary table 3. Multivariate logistic regression of MUI by BDCPP T with and without adjustment**

|  |  | Total | |  | Men | |  | Women | |  |
| --- | --- | --- | --- | --- | --- | --- | --- | --- | --- | --- |
|  |  | OR (95%CI) | p value |  | OR (95%CI) | p value |  | OR (95%CI) | p value |  |
| Adjust I | T 1 | 1 |  |  | 1 |  |  | 1 |  |  |
|  | T 3 | 0.88 (0.55, 1.40) | 0.5879 |  | 1.88 (0.50, 7.01) | 0.3489 |  | 0.77 (0.47, 1.26) | 0.2940 |  |
|  | T 3 | 1.02 (0.61, 1.71) | 0.9505 |  | 0.25 (0.02, 2.63) | 0.2496 |  | 1.10 (0.64, 1.89) | 0.7331 |  |
|  | p for trend | 1.00 (0.86, 1.16) | 0.9944 |  | 0.82 (0.51, 1.30) | 0.3901 |  | 1.02 (0.87, 1.19) | 0.8483 |  |
| Adjust II | T 1 | 1 |  |  | 1 |  |  | 1 |  |  |
|  | T 2 | 0.84 (0.52, 1.34) | 0.4619 |  | 1.33 (0.29, 6.11) | 0.7128 |  | 0.72 (0.43, 1.20) | 0.2075 |  |
|  | T 3 | 0.90 (0.53, 1.55) | 0.7087 |  | 0.18 (0.01, 2.29) | 0.1848 |  | 0.98 (0.56, 1.73) | 0.9542 |  |
|  | p for trend | 0.97 (0.83, 1.13) | 0.6610 |  | 0.71 (0.41, 1.26) | 0.2434 |  | 0.98 (0.84, 1.16) | 0.8314 |  |

**Supplementary table 4. Multivariate logistic regression of MUI by BCEP T with and without adjustment**

|  |  | Total | |  | Men | |  | Women | |  |
| --- | --- | --- | --- | --- | --- | --- | --- | --- | --- | --- |
|  |  | OR (95%CI) | p value |  | OR (95%CI) | p value |  | OR (95%CI) | p value |  |
| Adjust I | T 1 | 1 |  |  | 1 |  |  | 1 |  |  |
|  | T 3 | 0.74(0.46, 1.17) | 0.1995 |  | 0.69(0.19, 2.51) | 0.5776 |  | 0.75(0.46, 1.23) | 0.2527 |  |
|  | T 3 | 0.98(0.63, 1.53) | 0.9276 |  | 0.31(0.06, 1.56) | 0.1543 |  | 1.07(0.66, 1.72) | 0.7882 |  |
|  | p for trend | 1.01(0.86, 1.18) | 0.9109 |  | 0.67(0.40, 1.15) | 0.1469 |  | 1.05(0.88, 1.24) | 0.5999 |  |
| Adjust II | T 1 | 1 |  |  | 1 |  |  | 1 |  |  |
|  | T 2 | 0.72(0.45, 1.17) | 0.1814 |  | 0.47(0.11, 2.00) | 0.3079 |  | 0.76(0.45, 1.28) | 0.3069 |  |
|  | T 3 | 0.97(0.61, 1.54) | 0.8859 |  | 0.34(0.06, 1.87) | 0.2134 |  | 1.07(0.65, 1.77) | 0.7789 |  |
|  | p for trend | 1.01(0.85, 1.19) | 0.9421 |  | 0.67(0.36, 1.22) | 0.1864 |  | 1.05(0.88, 1.25) | 0.5962 |  |

**Supplementary table 5. Multivariate logistic regression of MUI by DBUP T with and without adjustment**

|  |  | Total | |  | Men | |  | Women | |  |
| --- | --- | --- | --- | --- | --- | --- | --- | --- | --- | --- |
|  |  | OR (95%CI) | p value |  | OR (95%CI) | p value |  | OR (95%CI) | p value |  |
| Adjust I | T 1 | 1 |  |  | 1 |  |  | 1 |  |  |
|  | T 3 | 1.12 (0.71, 1.78) | 0.6270 |  | 4.33 (0.48, 39.03) | 0.1915 |  | 0.99 (0.61, 1.60) | 0.9574 |  |
|  | T 3 | 0.83 (0.50, 1.39) | 0.4773 |  | 4.15 (0.42, 40.70) | 0.2218 |  | 0.73 (0.43, 1.26) | 0.2658 |  |
|  | p for trend | 0.97 (0.82, 1.14) | 0.7052 |  | 1.58 (0.78, 3.22) | 0.2055 |  | 0.93 (0.78, 1.10) | 0.3879 |  |
| Adjust II | T 1 | 1 |  |  | 1 |  |  | 1 |  |  |
|  | T 2 | 1.13 (0.71, 1.82) | 0.6025 |  | 2.93 (0.30, 28.59 | ) 0.3542 |  | 1.05 (0.64, 1.73) | 0.8559 |  |
|  | T 3 | 0.89 (0.53, 1.51) | 0.6666 |  | 1.97 (0.18, 21.59) | 0.5772 |  | 0.83 (0.47, 1.45) | 0.5154 |  |
|  | p for trend | 0.99 (0.83, 1.17) | 0.8839 |  | 1.22 (0.59, 2.56) | 0.5890 |  | 0.96 (0.80, 1.15) | 0.6752 |  |

**Supplementary figure 1. Spline curves fitting based on penalized spline method for the associations between OPEs and MUI.**


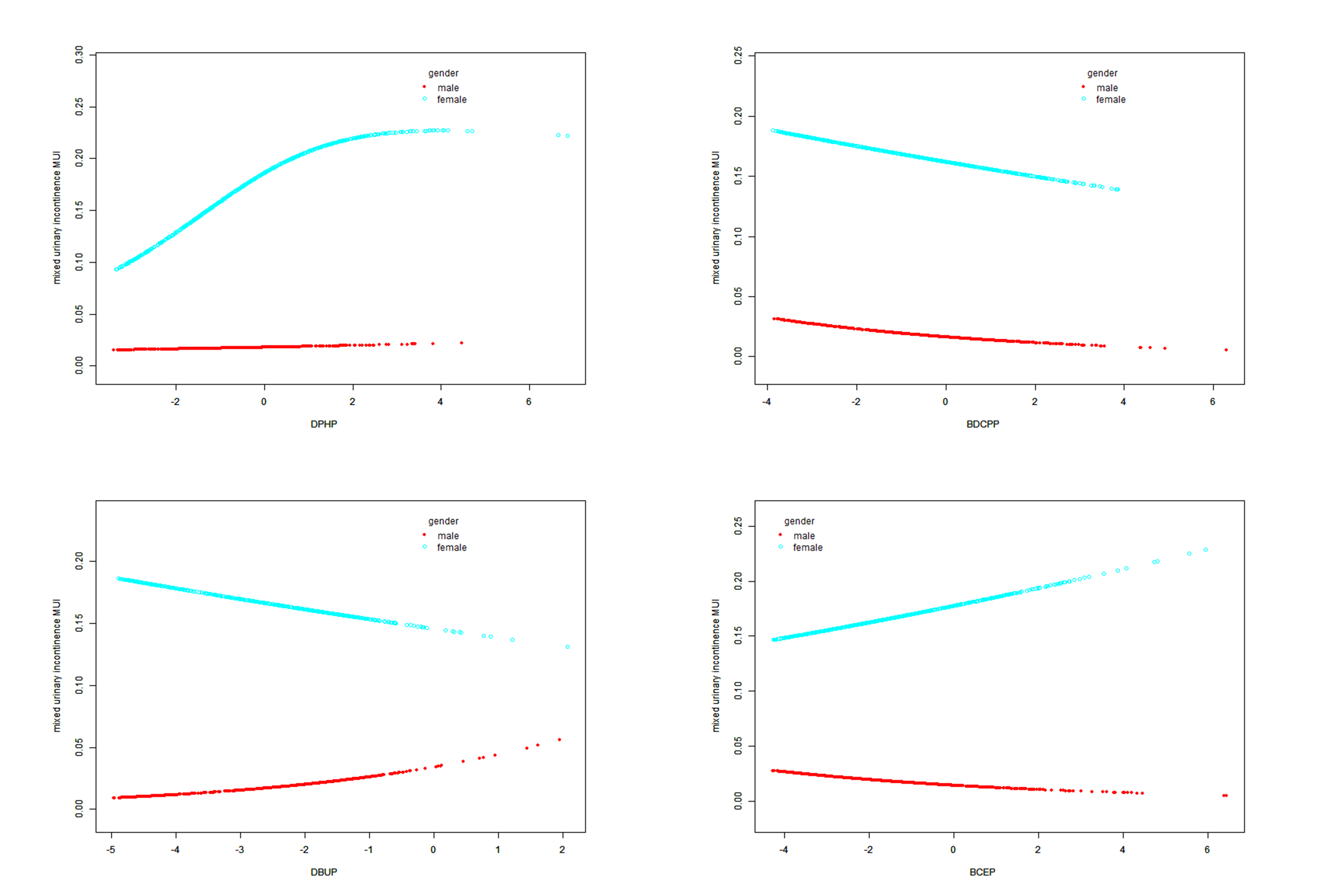

Supplement: Supplementary file 1 — (DOCX 2450 kb) [file 11356_2021_14153_MOESM1_ESM.docx]
